# A novel anti-HER2 anthracycline-based antibody-drug conjugate induces adaptive anti-tumor immunity and potentiates PD-1 blockade in breast cancer

**DOI:** 10.1186/s40425-018-0464-1

**Published:** 2019-01-21

**Authors:** Lucia D’Amico, Ulrike Menzel, Michael Prummer, Philipp Müller, Mélanie Buchi, Abhishek Kashyap, Ulrike Haessler, Alexander Yermanos, Rémy Gébleux, Manfred Briendl, Tamara Hell, Fabian I. Wolter, Roger R. Beerli, Iva Truxova, Špíšek Radek, Tatjana Vlajnic, Ulf Grawunder, Sai Reddy, Alfred Zippelius

**Affiliations:** 1 Cancer Immunology, Department of Biomedicine, University of Basel, Basel, Switzerland; 2 Department of Biosystems Science and Engineering, ETH Zürich, Basel, Switzerland; 3 NEXUS Personalized Health Technologies, ETH Zürich, Switzerland and Swiss Institute of Bioinformatics, Zürich, Switzerland; 4 NBE-Therapeutics Ltd, Hochbergerstrasse 60C, 4057 Basel, Switzerland; 5 Sotio s.a, Jankovcova 1518/2, 170 00 Prague 7, Czech Republic; 6 Institute of Pathology, University Hospital Basel, Basel, Switzerland; 7 Department of Cancer Immunology and Immune Modulation, Boehringer Ingelheim Pharma GmbH and Co. KG, Birkendorfer Str. 65, 88400 Biberach an der Riss, Germany; 8 Celonic AG, Eulerstrasse 55, 4051 Basel, Switzerland

**Keywords:** Antibody-drug conjugates, HER2-positive breast cancer, Anthracycline, Checkpoint inhibitor combination therapy

## Abstract

Increasing evidence suggests that antibody-drug conjugates (ADCs) can enhance anti-tumor immunity and improve clinical outcome. Here, we elucidate the therapeutic efficacy and immune-mediated mechanisms of a novel HER2-targeting ADC bearing a potent anthracycline derivate as payload (T-PNU) in a human HER2-expressing syngeneic breast cancer model resistant to trastuzumab and ado-trastuzumab emtansine. Mechanistically, the anthracycline component of the novel ADC induced immunogenic cell death leading to exposure and secretion of danger-associated molecular signals. RNA sequencing derived immunogenomic signatures and TCRβ clonotype analysis of tumor-infiltrating lymphocytes revealed a prominent role of the adaptive immune system in the regulation of T-PNU mediated anti-cancer activity. Depletion of CD8 T cells severely reduced T-PNU efficacy, thus confirming the role of cytotoxic T cells as drivers of the T-PNU mediated anti-tumor immune response. Furthermore, T-PNU therapy promoted immunological memory formation in tumor-bearing animals protecting those from tumor rechallenge. Finally, the combination of T-PNU and checkpoint inhibition, such as α-PD1, significantly enhanced tumor eradication following the treatment. In summary, a novel PNU-armed, HER2-targeting ADC elicited long-lasting immune protection in a murine orthotopic breast cancer model resistant to other HER2-directed therapies. Our findings delineate the therapeutic potential of this novel ADC payload and support its clinical development for breast cancer patients and potentially other HER2 expressing malignancies.

**Electronic supplementary material:**

The online version of this article (10.1186/s40425-018-0464-1) contains supplementary material, which is available to authorized users.

## Introduction

Epidermal growth factor receptor 2 (HER2) is amplified and overexpressed in about 20% of all breast cancer patients [1, 2]. To date, three HER2-specific agents have been approved by regulatory agencies for the treatment of breast cancer: trastuzumab, pertuzumab and ado-trastuzumab emtansine (T-DM1). Trastuzumab and pertuzumab are monoclonal antibodies (mAb) directed against the extracellular subdomain IV and II of HER2, respectively, which exert their therapeutic effect through inhibition of HER2 signaling and receptor dimerization as well as conferring effector function through their Fc domain [3]. T-DM1 is an antibody-drug conjugate (ADC) combining trastuzumab-mediated target (HER2)-specificity with the chemotherapeutic potency of the microtubulin polymerization inhibitor maytansinoid DM1 as payload, thus allowing for targeted drug delivery [4]. T-DM1 showed improved survival compared to trastuzumab and was approved by the U.S. Food and Drug Administration in 2013 [5, 6]. However, despite improved outcomes in many patients, tumors can develop various and complex resistance mechanisms [7]. This urges expansion of the therapeutic arsenal by development of drugs that are more potent and/or target novel pathways. In the ADC field, efforts are currently under way to develop site-specific conjugation technologies, novel compounds with increased cytotoxicity, and combination therapies with checkpoint inhibitors [8–10].

Here we tested a recently developed, novel HER2-targeting ADC composed of trastuzumab conjugated to a derivate of the highly potent anthracycline PNU-159682 through a non-cleavable peptide linker by sortase-mediated antibody conjugation (SMAC) technology [11, 12], hereafter called T-PNU. The SMAC technology allows for homogenous and stable ADC preparations with defined and favorable drug-to-antibody ratios and high in vitro and in vivo potency [11, 12].

PNU-159682 (3′-deamino-3″,4′-anhydro-[2″(*S*)-methoxy-3″(*R*)-oxy-4″-morpholinyl]) is a metabolite of nemorubicin (MMDX), and was found to be 700- to 2400-fold more potent than its parent drug in cultured human tumor cells. Importantly, ADCs based on PNU-159682 showed enhanced efficacy in in vivo tumor models compared to ADCs based on conventional payloads [12–14].

Anthracyclines have been used in anticancer therapy for more than 40 years. Doxorubicin became the most widely used anthracycline due to its low toxicity and potent anti-tumor activity against solid tumors [15, 16]. Despite generic immunomodulatory effects, evidence supports a role for anthracyclines in boosting a specific anti-tumor immune response by triggering a programmed, immunogenic variant of apoptosis known as immunogenic cell death (ICD) [17, 18]. The immunostimulatory activity of anthracyclines has been linked to their capacity to kill cancer cells upon activation of a multi-pronged adaptive stress response involving autophagy, the endoplasmic reticulum (ER) and type I interferon signalling. The ER stress response underlies the exposure of the ER chaperone calreticulin (CRT), HSP70 and HSP90 as well as the release of ATP and reactive oxygen release (ROS). Altogether, these signals released from dying cancer cells are known as damage/danger-associated molecular patterns (DAMPs) and are commonly used as ICD markers [19, 20].

Our group has extensively characterized the immunostimulatory properties of certain ADC payloads such as maytansinoid, ansamitocin P3 and dolastatins [21, 22]. Accordingly, we have recently shown that the ADC T-DM1 actively stimulates tumor-specific immune responses in breast cancer patients and HER2-expressing breast cancer models through DM1-mediated maturation of dendritic cells [9]. Here, we dissect the immune response of the novel ADC T-PNU in a syngeneic, murine breast cancer model overexpressing human HER2 and nonresponsive to both trastuzumab and T-DM1 treatments. We identified the immune-related properties of T-PNU, resulting in the stimulation of CD8 T cell immunity and protective immunological memory formation. Finally, T-PNU treatment was accompanied by an increased survival of most tumor-bearing animals when combined with checkpoint inhibitor therapies.

## Materials and methods

### Animals and tumor models

Balb/c animals were bred in the animal facility of the Department of Biomedicine, University of Basel, Switzerland. All animals were housed under specific pathogen-free conditions in accordance with Swiss federal regulations.

EMT6-hHER2 is a murine breast cancer cell line expressing human HER2 (hHER2). Human full-length HER2 cDNA was cloned into the transposon-based vector pPB-PGK-Puro and co-transfected into murine wild-type (WT) EMT6 cells together with the PiggyBac transposase expression vector pcDNA3.1/hyPB [23]. Then, cell pools stably expressing HER2 were selected in the presence of puromycin and individual clones were isolated by single-cell sorting using flow cytometry (FACS Aria II, BD Biosciences).

EMT6-hHER2, EMT6-WT and TS/A Thy1.1 murine breast cancer cell lines were cultured at 37 °C in complete media (DMEM supplemented with 2 mM L-glutamine, 100 μg/mL streptomycin, 100 U/mL penicillin, and 1 mM sodium pyruvate) containing 10% fetal bovine serum (FBS). To establish tumors, EMT6-hHER2 (10^6^), EMT6-WT (2.5 × 10^5^) and TS/A Thy1.1 (2.5 × 10^5^) cells in phosphate-buffered saline (PBS) were implanted into the mammary gland of female mice. Tumor measurements were performed three times a week with a caliper and volumes were calculated using the following formula: V = ½ {length (mm) × [width (mm)]^2^}.

### In vivo tumor models and efficacy studies

EMT6-hHER2 and EMT6-WT cells were injected into the mammary gland of female BALB/c mice (8–10 weeks old). Once tumors reached an average volume of 80 mm^3^ (day0), mice were treated with T-PNU (1 mg/kg) and/or α-mouse PD1 (12.5 mg/kg) (RPM1–14, rat IgG2a, Biox Cell), T-DM1 (15 mg/kg), trastuzumab (20 mg/kg) or mitoxantrone (2 μg/kg) or as indicated in the figures or legends. Tumor volume was measured three times per week as 1 alone or in combination with α-PD1 at day 0, 2, 4, 7. For FACS analysis and CD45-positive cell isolation, T-PNU, trastuzumab or T-DM1 were given as single treatment once the tumors reached 80 mm^3^ at the concentration indicated above. Animals were euthanized 10 days after treatments, when tumors in the T-PNU cohort reached a volume of 30 mm^3^ suitable for tumor harvest and cell processing. For T cell depletion experiments, α-CD8 depleting antibodies (53–6.72, rat IgG2a, Bio X Cell) were given at day − 2, 0, and once a week for the next 4 weeks at 10 mg/kg. Mice were euthanized once tumors reached a volume of 1200 mm^3^. For re-challenge experiments, T-PNU-cured animals received EMT6-hHER2 (10^6^), EMT6-WT (2.5 × 10^5^) or T/SA Thy1.1 (2.5 × 10^5^) tumor cells by mammary fat pad injection and orthotopic tumor growth was measured for a further 70 days.

### Quantification of ICD properties of T-PNU in vitro

EMT6-hHER2 cells were treated with 150 ng/mL of T-PNU for 48 and 72 h and compared to idarubicine (20 μg/mL) and nonimmunogenic ultraviolet B (UVB)-treated cells. The percentage of early (annexin V^+^/DAPI^−^) and late (annexin V^+^/DAPI^+^) apoptotic cells and necrotic cells (annexin V^−^/DAPI^+^) was determined by flow cytometry. To quantify HSP70, HSP90 and CRT expression on the cell surface, EMT6-hHER2 cells were treated with 150 ng/mL of T-PNU for 48 and 72 h or with idarubicine (20 μg/mL). HSP70 (R&D Systems, #RD-MAB1663), HSP90 (Enzo Life Sciences, #ADI-SPA-830-F) and CRT antibody (Abcam, #ab2907) were used for flow cytometry.

The expression of these molecules was then detected by flow cytometry on live (annexin V^−^/DAPI^−^) and non-permeabilized (annexin V^+^/DAPI^−^) cells. Next, ROS production was determined in the presence or absence of ROS inhibitors, N-acetyl-L-cysteine (NAC, 5 mM) and reduced L-glutathione (GSH, 5 mM), in EMT6-hHER2 cells treated with 150 ng/mL T-PNU or idarubicine for 48 h. To quantify the intracellular or extracellular ATP levels, EMT6-hHER2 cells were exposed to T-PNU (48 and 72 h), idarubicine and UVB (24 h respectively). Quantification of ATP was assessed using an ATP bioluminescent assay kit based on luciferin-luciferase conversion (Sigma Aldrich, #FL-AA).

Finally, the release of HMGB1 into culture supernatants after T-PNU incubation of EMT6-hHER2 was determined by enzyme linked immunosorbent assay (HMGB1 ELISA–IBL International, #ST51011).

### Immunofluorescence

Cells were collected, washed twice with PBS and fixed in 4% paraformaldehyde for 20 min. After the washing with PBS, cells were incubated in 1% BSA in PBS for 1 h and subsequently incubated with CRT (Enzo Life Sciences, 1:100), HSP70 (R&D, 1:100) or HSP90 (Enzo Life Sciences, 1:100) antibody in PBS overnight at 4 °C. Cells were washed with PBS and incubated with AlexaFluor488 goat anti-mouse (Molecular Probes, 1:200) IgG1 (CRT and HSP90) or with AlexaFluor488 goat anti-mouse IgG2A (HSP70) secondary antibody (Molecular Probes, diluted 1:200) for 1 h at 4 °C. Cells were washed with PBS and incubated with Wheat Germ Agglutinin, Alexa Fluor 594 Conjugate (Invitrogen, 5 μg/ml) for 10 min at room temperature. Then the cells were washed with PBS and mounted on slides with ProLong Gold antifade reagent with DAPI (Molecular Probes) using StatSpin Cytofuge. Cells were analysed under a DMI 6000 inverted Leica TCS AOBS SP5 tandem scanning confocal microscope and an × 63 oil immersion objective.

### Tumor digestion

Tumors were mechanically digested with accutase (PAA), collagenase IV (Worthington), hyaluronidase (Sigma), and deoxy-ribonuclease type IV (Sigma). Single-cell suspensions were then prepared and stained for flow cytometry analysis (FACS) or CD45-positive cell enrichment.

### Flow cytometric analysis

Cell suspensions were stained for FACS analysis with the following antibodies: α-CD3-PE (BD Biosciences), α-CD8-BV605 (BD Biosciences), α-CD45-PercP (BD Biosciences), α-CD45-PE (BD Biosciences), α-CD11b-FITC (BioLegend), α-CD11c-FITC (BioLegend), α-CTLA4-PE (BD Biosciences), α-granzyme B-Alexa fluor 647 (BD Biosciences), α-IFNγ-APC (BD Biosciences), α-KI67-BV421 (BD Biosciences), α-TIM3-PE (eBioscience), α-TIGIT-PE (BD Biosciences), α- TNFα-PE (BD Biosciences), α-IL17r-PE (BD Biosciences), and the corresponding isotype control (mouse IgG1, Southern Biotech). The live/dead fixable near-infrared dye (Invitrogen) was used to exclude dead cells. Intracellular fixation and permeabilization buffers from eBioscience were used for cytokine staining.

Acquisition was performed on FACS BD Fortessa and the dedicated software CellQuest (BD Biosciences). Data was analyzed with FlowJo 7.5.5 software (TreeStar Inc).

### Immunohistochemistry analysis

FFPE tissue blocks were cut at 4 μm. Immunohistochemistry was performed using anti-HER-2/neu rabbit monoclonal antibody (clone 4B5, prediluted, Ventana Medical Systems Inc.). The analyses were performed on the BenchMark XT automated immunostainer using the OptiView detection system (Ventana Medical System Inc., Tuscon, AZ). Membranous staining was considered positive. The intensity of staining was scored as weak, moderate, and strong by our pathologist Dr. Tatjana Vlajnic at the University Hospital in Basel.

### Isolation of CD45-positive cells from tumors

CD45-positive cells were isolated by magnetic bead enrichment. First, single-cell tumor suspensions were stained with α-CD45-PE antibody (BD) for 15 min with FACS buffer. Then, PE enrichment was performed with α-PE magnetic beads using the EasySep Mouse PE Positive Selection Kit (STEMCELL Technologies). Purity was confirmed by flow cytometry (> 95%). Cells were lyzed in TRIzol and stored at − 80 °C for RNA extraction.

### RNA-sequencing

RNA was isolated using the TRIzol Plus RNA Purification Kit (Life Technologies) according to the manufacturer’s protocol yielding high quality RNA (average RNA integrity number > 8). RNA-seq libraries were prepared using the Truseq stranded mRNA Library Prep Kit from Illumina with 200 ng input material. Samples were run on an Illumina NextSeq 500 using the NextSeq 500/550 High Output v2 kit (75 cycles). RNA-seq raw and processed data is available on the NCBI GEO site under the accession number GSE120888.

### Preprocessing, read mapping and quality control

Starting from the raw fastq files (1x81bp), the reads were mapped against the mouse reference genome and simultaneously soft-clipped using the STAR aligner (version 2.4.2a). Standard quality control (Picard, RSeQC, QoRTs) was run to assess the quality of the resulting alignments. Subsequently, strand-specific (reversely stranded) read counting was performed using featureCount (subread, version 1.5.0). The library size of the 24 samples was of reasonably good quality. To avoid technical artifacts the data were filtered such that genes were removed where the average read depth over all samples was < 5 counts. Transcripts were further mapped to gene symbols and ENTREZID’s using the org.Mm.eg.db package version 3.4.0. Gene expression similarity between samples, within, and between groups were determined in order to identify and eliminate possible outliers.

### Differential gene expression analysis

Differential expression analysis (DEA) was performed between the different experimental conditions. To this end, the read counts per gene were modeled using the generalized linear regression of the negative binomial family, as implemented in the DESeq2 package [24] of the R environment for statistical computing [25]. Thresholds for both, the false discovery rate FDR < 0.01 and the expression fold-change > 2 or < − 2 were applied.

### Gene set analysis

Gene set analyses (GSA) for all comparisons was performed on the Molecular Signature Database (MSigDB) curated gene set collection C2 from the Walter + Eliza Hall Institute of Medical Research, in particular BIOCARTA (217 gene sets), KEGG (186 gene sets), PID (196 gene sets) and REACTOME (674 gene sets). Competitive enrichment analysis was performed on 1297 curated gene sets, where *p*-values are estimated by random rotations as implemented in the fry() function of the limma package [26]. Gene sets were called significantly differentially regulated if FDR < 0.05 and labeled “up”, “down” or “mixed” if the genes in the set expressed an overall positive, negative or mixed fold-change characteristics, respectively.

### Gene set network analysis

For the network analysis, gene sets were retained with a false discovery rate (FDR) < 0.05 and less than 200 gene members. Candidate gene set networks were constructed from the pairwise adjacency matrix based on a minimum Jaccard index of 0.2. Nodes with degree < 3 were discarded. For network refinement, larger clusters were split into sub-graphs with the help of a community detection algorithm based on the edge betweenness centrality score as implemented in the igraph package {http://igraph.org} in R.

### Clonotype analysis

Immunoglobulin CDR3 information was extracted using the MiXCR software package for bulk RNA-seq data using the recommended parameter settings [27]. The analysis was focused on the TCRβ repertoire as the higher diversity is a close indicator for unique T cell clones. Normalization of unique TCRβ clone counts by sequence abundance (total read count) was used as proxy for clonal expansion.

### Statistics

Tumor samples and survival curves were analyzed with GraphPad Prism 7.0 software using ANOVA and Dunn’s multiple comparison test. Scatter dot plots are depicted as mean with SEM.

## Results

### Generation of a murine EMT6-hHER2 breast cancer cell line

In order to investigate T-PNU anti-tumor efficacy in vivo, we first established a murine breast cancer cell line overexpressing hHER2 following stable transposition of a hHER2 expression cassette into the murine breast cancer cell line EMT6. Human HER2 protein expression was confirmed by flow cytometry and an EMT6 clone expressing homogeneous levels of hHER2 was isolated by single-cell sorting (Additional file 1: Figure S1A). Immunohistochemistry analysis of EMT6-hHER2 tumors further confirmed a strong expression of human HER2 (Additional file 1: Figure S1B). To demonstrate T-PNU mediated cellular cytotoxicity, a cell proliferation assay was performed by incubating 10^4^ EMT6-hHER2 cells with a dilution series of site-specifically conjugated T-PNU [12] or T-DM1. While EMT6-hHER2 cells showed sensitivity towards T-PNU treatment, they were unresponsive to T-DM1. EMT6-WT cells incubated with T-PNU or T-DM1 were, however, not affected by the treatments confirming T-PNU specificity to the HER2-antigen in vitro (Additional file 1: Figure S1C).

### T-PNU strongly affects EMT6-hHER2 breast tumor growth in vivo

**Fig. 1:**
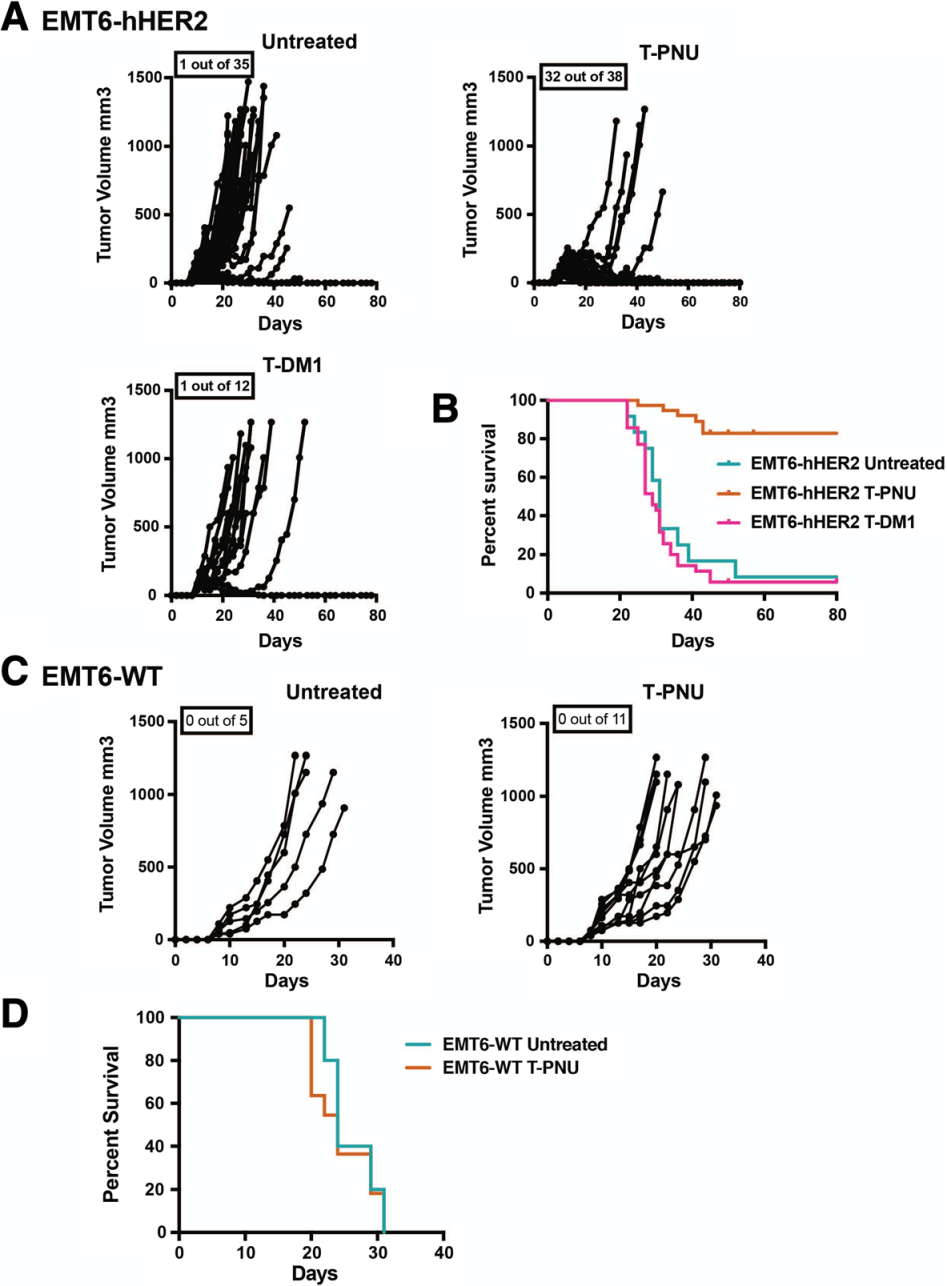
T-PNU strongly affects tumor growth in hHER2 orthotopic breast cancer model unresponsive to T-DM1 treatment. **a,** Therapeutic response of EMT6-hHER2 tumor-bearing mice following treatment with T-PNU (1 mg/kg, 2x) and T-DM1 (15 mg/kg, 2x). Mice were treated when tumors reached an average tumor volume of 80 mm^3^ and were euthanized at 1200 mm^3^. Pooled data from at least three independent experiments (n = number of cured mice out of treated animals as indicated). **b**, Overall survival curves showing results of **a**. **c**, Therapeutic response of EMT6-WT tumor-bearing mice following treatment with T-PNU (1 mg/kg, 2x). Pooled data from two independent experiments (n = number of cured mice out of treated animals as indicated. **d**, Overall survival curves showing results of **c**

To evaluate T-PNU efficacy in vivo we injected 10^6^ EMT6-hHER2 cells orthotopically into the mammary fat pads of immunocompetent Balb/c mice. Mice with established tumors were treated i.v. with T-PNU or T-DM1. While T-PNU treatment resulted in a strong anti-tumor response with more than 80% of cured animals (Fig. 1a, b), treatment with trastuzumab alone (Additional file 1: Figure S2) or T-DM1 (Fig. 1a, b) did not prevent tumor growth, supporting a prominent role of the payload component PNU in the suppression of tumor progression. No significant anti-tumor effect was observed with the free PNU payload in vivo, underlining the importance of targeting the cytotoxic drug by antibody targeting to the cellular receptor HER2 for efficient internalization (Additional file 1: Figure S2). In addition and in line with our in vitro observations, EMT6-WT tumor-bearing animals did not respond to T-DM1 or T-PNU treatment (Fig. 1c, d).

In summary, the in vivo results demonstrate that a low dose of the novel ADC T-PNU is able to effectively treat even trastuzumab and T-DM1 resistant breast cancer cells.

### T-PNU induces immunogenic cell death in breast cancer

**Fig. 2:**
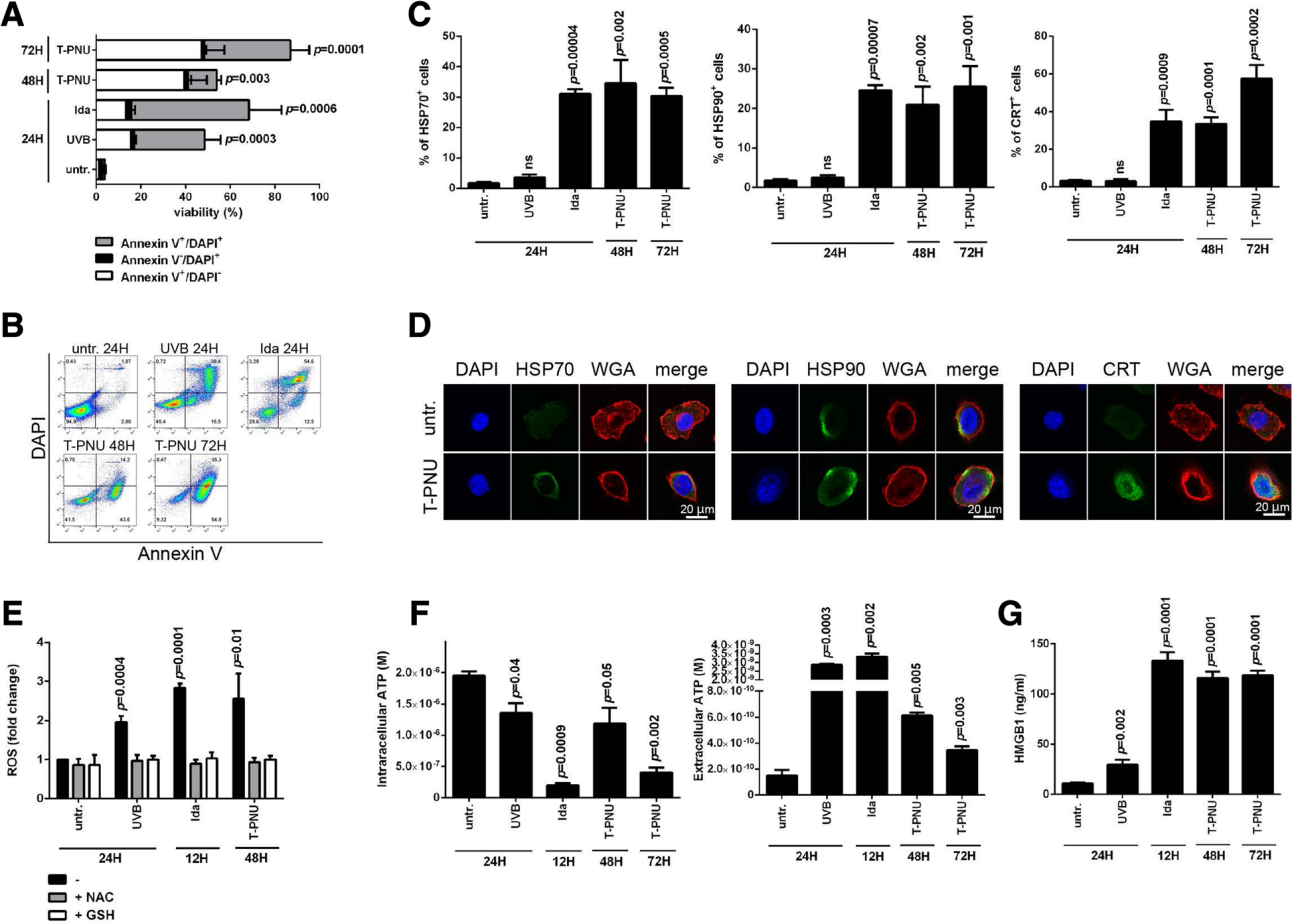
T-PNU displays immunogenic cell death properties. **a**, EMT6-hHER2 cell death kinetics induced by T-PNU. Percentage of early (annexin V^+^/DAPI^−^) and late (annexin V^+^/DAPI^+^) apoptotic cells and necrotic cells (annexin V^−^/DAPI^+^) was determined by flow cytometry. Data presented as mean ± SD for 3 independent experiments. **b**, Representative dot plots of flow cytometry analysis of cell death induced by T-PNU, idarubicine and UVB radiation at indicated time points. **c**, T-PNU induction of HSP70, HSP90 and CRT on EMT6-hHER2 cells. UVB treated cells are used as controls. Experiments were performed in triplicates. **d**, Representative immunofluorescence staining of HSP70, HSP90 and CRT on EMT6-hHER2 following T-PNU treatment. The presence of indicated markers on the cell surface was verified by confocal microscopy. **e**, T-PNU induces oxidative stress and production of reactive oxygen species (ROS). ROS were stained by CellRox Deep Red reagent and detected by flow cytometry. Experiments were performed in triplicates. **f**, Quantification of intracellular or extracellular ATP levels in EMT6-hHER2 cell line exposed to T-PNU, idarubicine and UVB. Data presented as mean ± SD for 3 independent experiments. **g**, HMGB1 release into EMT6-hHER2 culture supernatants induced by T-PNU. The compiled results of a total of 3 experiments are shown

We next asked whether the tumor-targeted delivery of the potent anthracycline PNU payload would show immunogenic properties. To address this, we quantified the release of various DAMPs after exposure to T-PNU. After the identification of the optimal dose of T-PNU capable of promoting cell death in vitro (Fig. 2a, b), we measured HSP70, HSP90 and calreticulin (CRT) release in the cell culture supernatant after incubation of EMT6-hHER2 cells with T-PNU compared to ICD-inducing idarubicine (positive control) and nonimmunogenic UVB (negative control)-treated cells. As expected, the percentage of HSP70-, HSP90- and CRT-positive cells increased after incubation with T-PNU and idarubicine (Fig. 2c, d). Moreover, we addressed the ability of T-PNU to induce the production of ROS and to release ATP and HMGB1 in EMT6-hHER2 cells. Accordingly, both ROS production and accumulation of extracellular ATP and HMGB1 was increased after incubation of EMT6-hHER2 cells with T-PNU and idarubicine (Fig. 2e-g).

Altogether, the identification of elevated key ICD markers in EMT6-hHER2 cultures after T-PNU exposure strongly supports the ICD inducing properties of the novel compound and corroborates the current notion of the anthracycline payload’s ability to activate the immune system.

### T-PNU shows immunomodulatory properties in a syngeneic breast cancer model

**Fig. 3:**
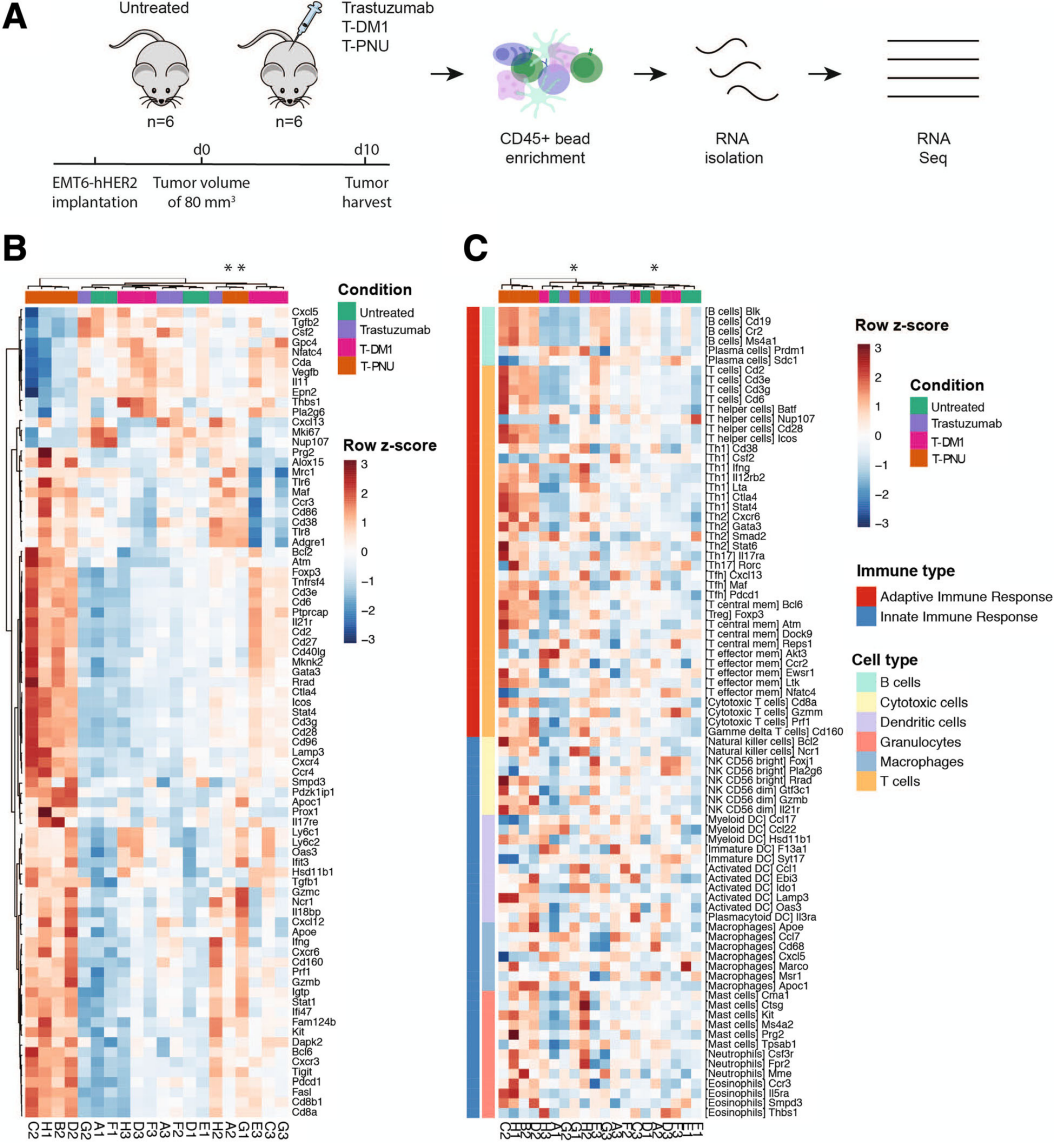
T-PNU induced tumor control involves immune responses. **a**, Experimental design of RNA-sequencing of CD45^+^ immune cells after treatment: Four cohorts of EMT6-hHER2 tumor-bearing mice (*n* = 6) received following treatments: (1) untreated, (2) trastuzumab (20 mg/kg, 1x), (3) T-DM1 (15 mg/kg, 1x), and (4) T-PNU (1 mg/kg, 1x). Tumors were isolated when reached an average of 30 mm^3^ and CD45-positive cells were isolated with magnetic beads selection. Extracted RNA was subjected to RNA-sequencing. **b**, Heatmap of a customized immune-specific gene panel. **c**, Heatmap of an immune phenotype gene panel including adaptive and innate immune response. Asterisks denote low-responding T-PNU samples

Due to the potential induction of ICD, we evaluated a therapeutic relevance for T-PNU mediated immune modulation of the tumor microenvironment. In order to identify the transcriptional pathways regulating the T-PNU response in the tumor environment, we performed RNA-sequencing analysis of CD45^+^ tumor-derived cells from untreated control animals and tumor-bearing animals treated with trastuzumab, T-DM1 or T-PNU (Fig. 3a, see *methods*). In a multi-dimensional scaling plot (Additional file 1: Figure S3A), gene expression analysis from responding T-PNU samples clustered clearly apart from non-responding untreated control, trastuzumab and T-DM1 treated tumors suggesting fundamental changes in gene expression within the intra-tumoral immune cell population of tumors treated with T-PNU. T-PNU responding samples also formed a separate cluster in a gene-expression heatmap showing the top 1000 differentially expressed genes (DEGs, Additional file 1: Figure S3B). To specifically investigate changes of immune-related genes, we focused on two gene lists; a first one including important immune-related genes and the second attributing genes to immune phenotypes [27–29]. The first allowed us to identify clear immune-related signatures for the four high-responding T-PNU samples with upregulated gene expression in 66 out of 80 selected immune genes (Fig. 3b). The same T-PNU samples also clustered in the phenotype analysis indicating a strong adaptive immune response with particular T cell involvement (Fig. 3c).

In summary, gene expression analysis revealed fundamental changes in the T-PNU treatment-responsive tumor samples at the immunogenomic level.

### T-PNU induced tumor control requires adaptive immunity

**Fig. 4:**
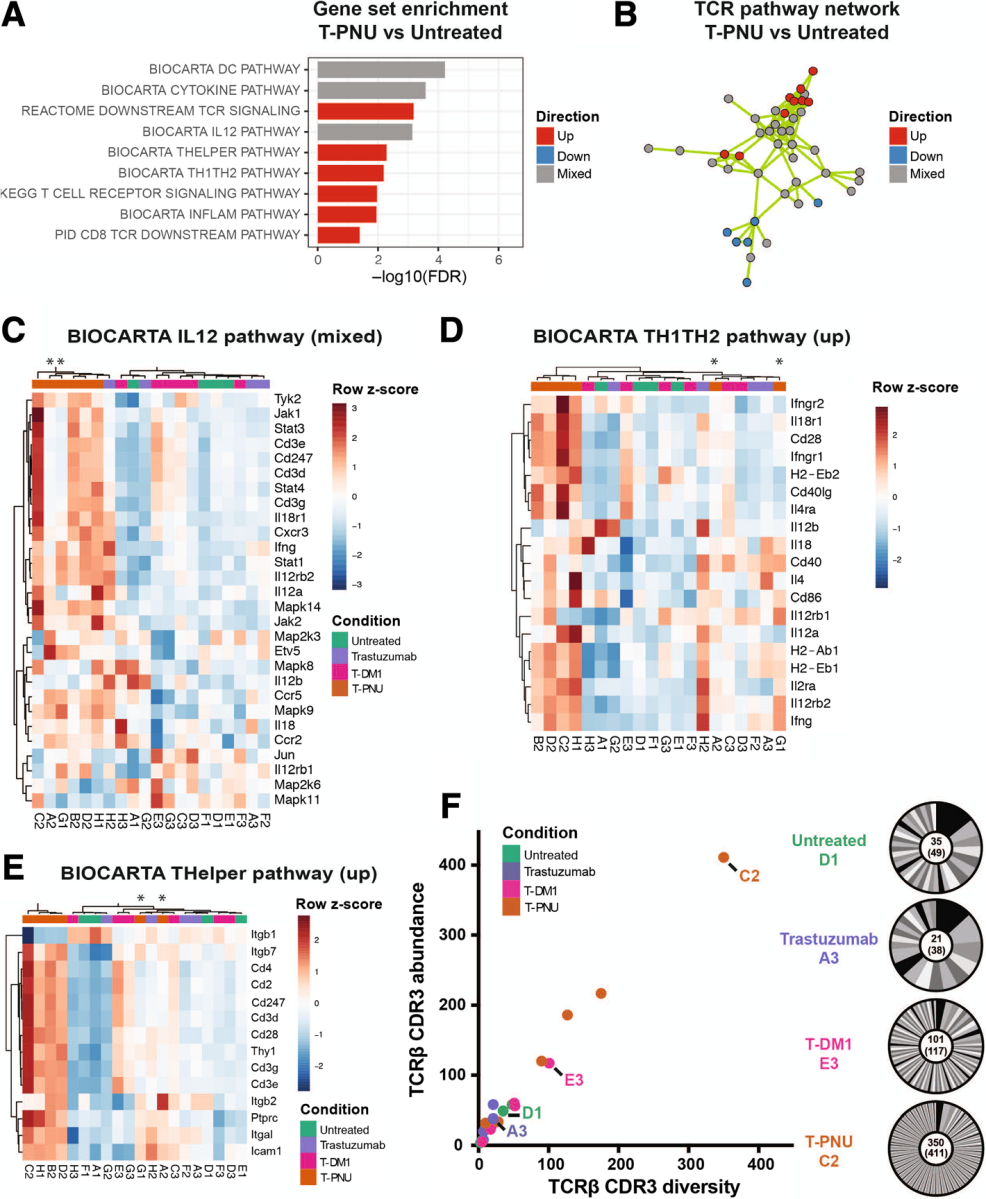
The T-PNU anti-tumor response consists of infiltrating and activated T cells. **a**, Gene set enrichment analysis of gene sets involved in T cell activation. Depicted is T-PNU vs. untreated. FDR: false discovery rate. **b**, Network cluster of gene sets with overlapping genes with TCR pathway function. The network includes 22 gene sets and 5448 genes (see Additional file 1: Table S1). **c-e,** Heatmaps of selected BIOCARTA THELPER, IL12 and TH1TH2 pathway gene sets, respectively (see Additional file 1: Figure S5 and S6 for more gene set heatmaps). **f**, MiXCR TCRβ CDR3 clonotype analysis. TCRβ CDR3 diversity (unique clones) plotted against TCRβ CDR3 abundance (read count). Donut plots depict clonotype distribution for one selected sample per cohort

We next performed gene set analysis (GSA) and found that T cell and inflammatory gene sets were upregulated upon T-PNU treatment compared to the untreated control after functional gene set selection (Fig. 4a) and in a network analysis based on genes shared between gene sets (Fig. 4b, Additional file 1: S6A, Additional file 1: Table S1, S2, see *methods*). Thus, at the functional level, BIOCARTA TH1TH2, THelper, CD8 TCR downstream and inflammatory pathways were positively associated with T-PNU treatment response, while genes in BIOCARTA IL12, dendritic cell (DC) and cytokine pathways were partially up- and downregulated. Within these pathways the T-PNU samples formed a distinct cluster separate from untreated, trastuzumab and T-DM1 treated samples and showed a Th1-driven response with the upregulation of IL12α, IFNγ, STAT4, IL12Rβ2, IL18R1, CXCR3, CCR5 and downregulation of Th2-associated chemokines IL6 and IL10 (Fig. 3b, Fig. 4c-e, Additional file 1: Figure S4 and S5B-D). Lastly, using MiXCR, we extracted complementarity determining region (CDR3) clonotype information from RNA-seq data [27] and found that T-PNU samples have higher numbers of infiltrating T cells based on TCRβ CDR3 occurrence (Fig. 4f). The T-PNU cohort contained on average almost 5 times more unique TCRβ CDR3s than non-T-PNU samples (130.0 ± 124.0 vs 28.0 ± 27.4 CDR3s, respectively). The numbers of unique TCRβ CDR3 clones (diversity, clone count) correlated with the numbers of total CDR3 transcripts (sequence abundance, read count) detected per sample suggesting that minimal clonal expansion occurred in the T-PNU treated cohort. Thus, while in vitro and in vivo data indicated HER2-dependent effects (Additional file 1: Figure S1B, S2), T cell responses are oligoclonal on a repertoire level and show no clonal focus to a particular target. Overall, the higher number of clonally diverse infiltrating T cells highlights the importance of the T cell response for tumor protection.

To further validate the RNA-seq analysis, we used flow cytometry to analyze the intra-tumoral immune cell subsets between the different treatment arms. After T-PNU treatment CD8 T cells were significantly increased in numbers and exhibited a higher functionality with increased expression of Granzyme B, Ki67, CTLA-4 and TIGIT, which were also found to be upregulated within the RNA expression data (Additional file 1: Figure S6A, B). Furthermore, CD8 T cells produced increased levels of TNFα and IFNγ in T-PNU treated animals supporting a crucial role for CD8 T cells in T-PNU-mediated tumor rejection (Additional file 1: Figure S6A, B).

Overall, we show on a transcriptomic, clonal and proteomic level the important role T cells play in the T-PNU anti-tumor response, which relies on increased number of oligoclonal T cells sharing Th1 as well as activated CD8 T cell signatures.

### CD8 T cells establish T-PNU mediated anti-tumor protection

**Fig. 5:**
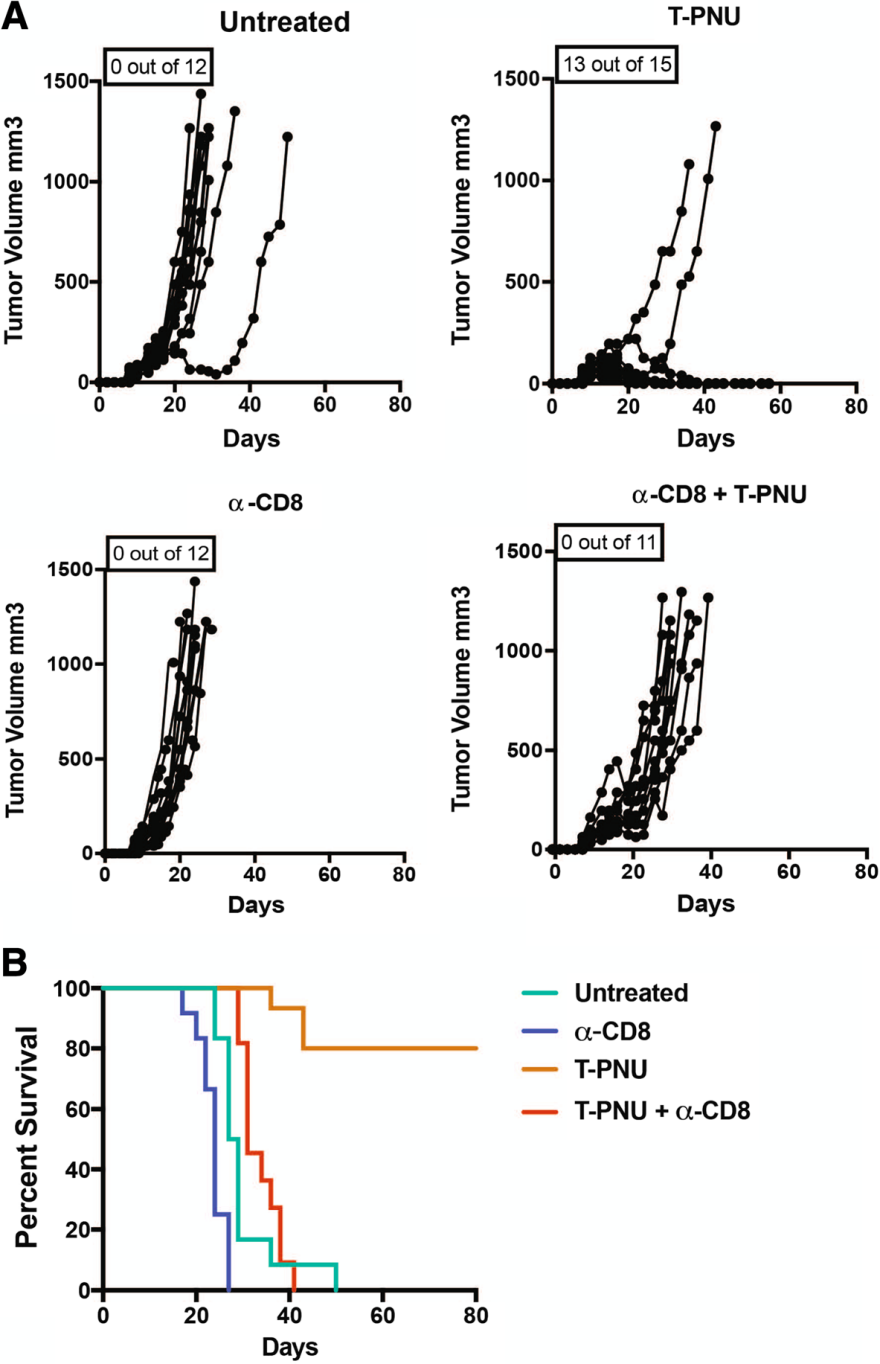
T-PNU anti-tumor protection is CD8 T cell-dependent. **a**, Tumor growth curves of EMT6-hHER2 tumor-bearing animals upon treatment with T-PNU (1 mg/kg, 2x), α-CD8 (10 mg/kg), or α-CD8 followed by T-PNU treatment, *P*-value < 0.0001 *** (Gehan-Breslow-Wilcoxon Test). **b**, Overall survival curves of **a**

Next, we tested the functional role of CD8 T cells for T-PNU therapeutic efficacy by depleting CD8 T cells using neutralizing anti-CD8 antibodies in tumor re-challenge experiments. The co-administration of neutralizing anti-CD8 antibodies severely reduced T-PNU anti-tumor protection and overall survival (Fig. 5a,b) suggesting that T-PNU activates an anti-tumor immunity an suppresses tumor growth through the recruitment of CD8 positive T cells at tumor site which are actively involved in tumor clearing.

### T-PNU ADC induces long-lasting tumor-type specific anti-tumor immunity

**Fig. 6:**
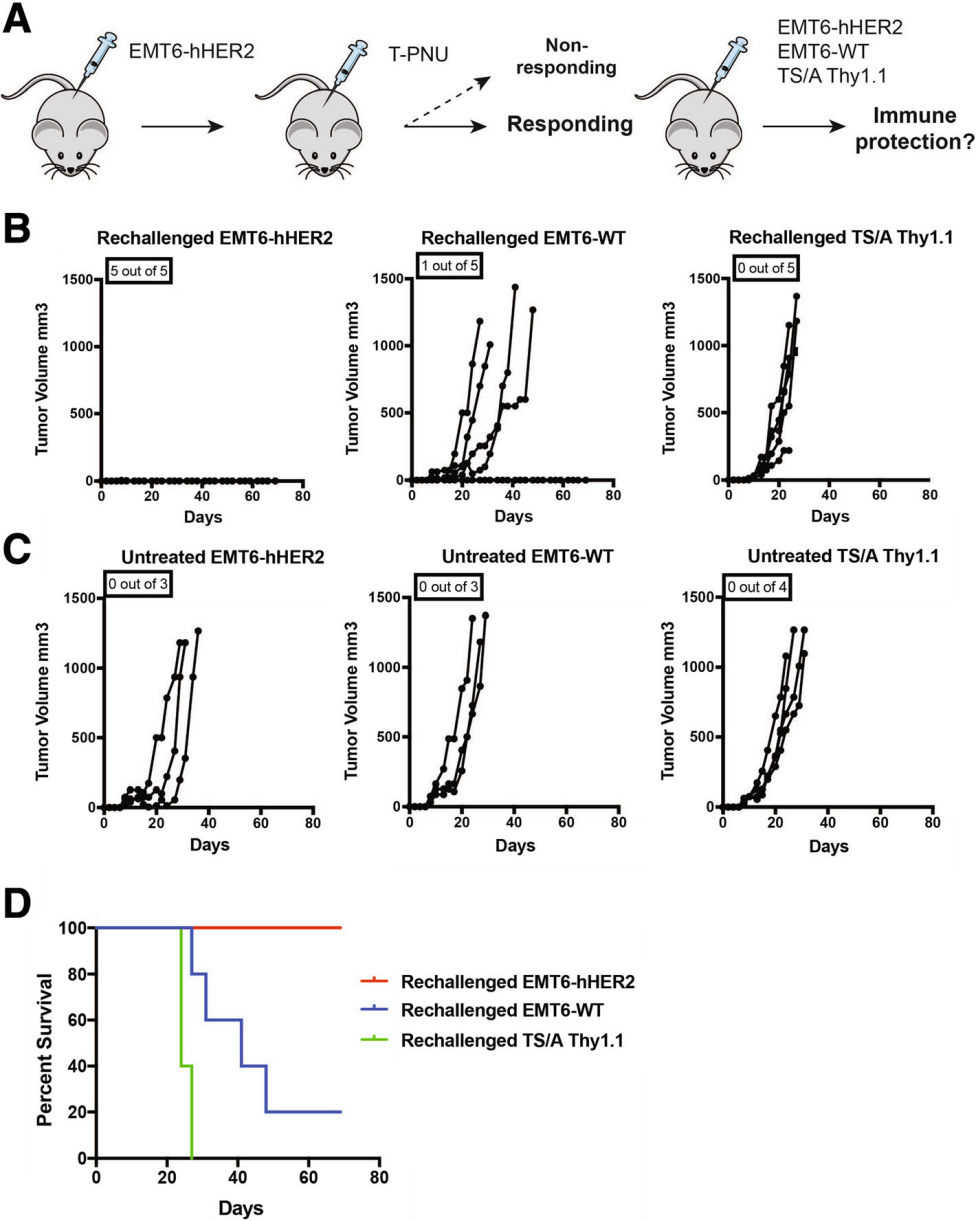
T-PNU promotes long-lasting immune protection. **a**, Outline of a representative rechallenge experiment. **b**, EMT6-hHER2 tumor-free animals after T-PNU treatment were rechallenged with EMT6-hHER2, EMT6-WT and TS/A Thy1.1 cancer cells. P-value < 0.0005 *** (Gehan-Breslow-Wilcoxon Test). **c**, Tumor growth of untreated controls. **d**, Overall survival curves of **b** and **c**

To further validate the specificity of the anti-tumor immunity and associated T cell responses upon T-PNU treatment, we next re-challenged T-PNU ADC treated animals with complete tumor regression by injection of the same tumor cells expressing hHER2, i.e. EMT6-hHER2, or the parental EMT6-WT or another murine breast cancer cell lineTS/A Thy1.1, both of which not expressing the human HER2 antigen (Fig. 6a). While EMT6-WT and TS/A Thy1.1 re-challenged mice rapidly developed tumors (Fig. 6b-d), EMT6-hHER2 re-challenged animals were completely protected from tumor development (Fig. 6b, d). This finding clearly demonstrates that the anti-tumor immunity induced by T-PNU ADC treatment is largely specific for the original hHER-2 expressing tumor cell line cleared upon T-PNU treatment, but not for other breast cancer cell lines. This indicates that T-PNU treatment led to the formation of a specific immunological memory.

### T-PNU induced tumor infiltration by CD8^+^ T cells sensitizes breast cancer to immune checkpoint therapy

**Fig. 7:**
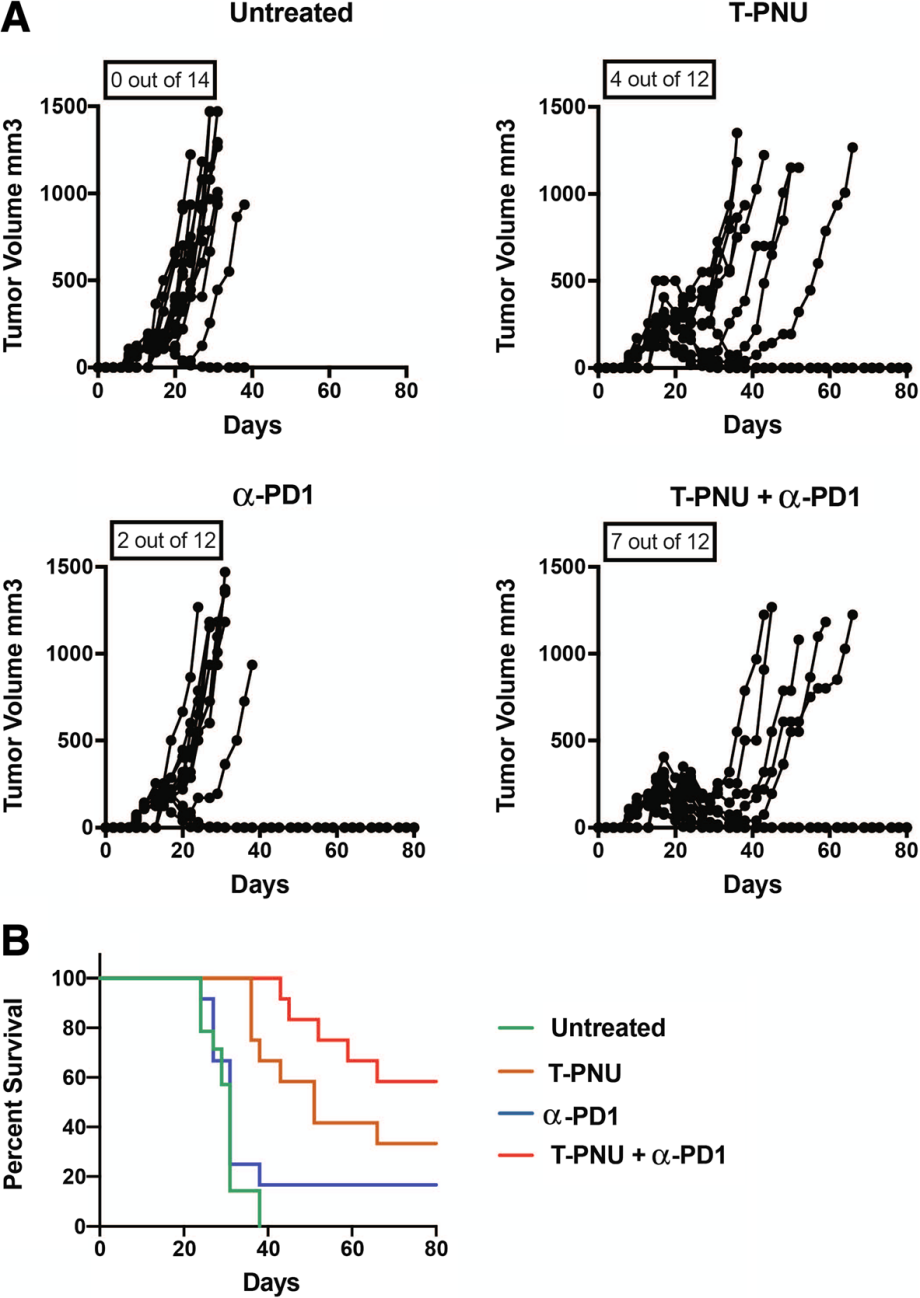
T-PNU treatment in combination with checkpoint inhibitor therapy. **a**, Therapeutic response of EMT6-hHER2 animals with a tumor volume of 150–200 mm^3^ following treatment with T-PNU (1 mg/kg, 2x) alone, α-PD1 alone (12.5 mg/kg) or their combination (T-PNU + α-PD1) *p*-value< 0.0001*** (Gehan-Breslow-Wilcoxon Test) in between all the survival curves. **b,** Survival curves of **a**

Our data highlight a critical role of CD8^+^ T cells for T-PNU mediated tumor control. In addition, T-PNU strongly activates T cells inducing an increased IFNγ up-regulation. Since IFNγ is known to induce PD-L1 expression, we assessed potential therapeutic benefits of a combination therapy consisting of T-PNU and PD1 blocking antibodies. Of note, our previous results showed high tumor clearance with T-PNU treatment with a tumor size of 80 mm^3^. In order to better study the effects of combination efficacy, we here allowed tumors to grow to a size of 150–200 mm^3^. Once tumors reached this volume, animals were treated with T-PNU and α-PD1 blocking antibodies. While α-PD1 or T-PNU therapies were largely ineffective at controlling these larger tumors, the combination of T-PNU with α-PD1 signicantly increased the percentage of cured animals (Fig. 7a, b), which provides the rationale for a novel combined strategy in a clinical setting.

## Discussion

Targeted cytotoxic therapies, such as ADCs, which specifically recognize tumor-associated surface antigens (TAA), have become an invaluable tool within current anti-cancer medicines. However, despite the very promising therapeutic efficacy of ADCs armed with microtubule-destabilizing warheads, resistance, either intrinsic or acquired, remains a clinical challenge in the management of patients. Important factors include, but are not limited to, low TAA expression, poor internalization and/or inefficient trafficking of the ADC complex, as well as expression of drug efflux pumps and/or multi-drug resistance transporters [30]. One important mechanism of T-DM1 resistance has recently been identified and traced to impaired lysosomal proteolytic activity in T-DM1 resistant cancer cells derived from HER2-positive patients [31].

To further improve ADC efficacy and to overcome resistance mechanisms, considerable effort has been made to develop HER2-targeting ADCs with novel, highly potent cytotoxic payloads that act via mechanisms beyond microtubule disruption. Here, we describe for the first time the therapeutic activity of a trastuzumab-based, HER2-targeting ADC incorporating a derivative of a novel anthracycline payload PNU-159682 (short T-PNU) in an orthotopic, syngeneic murine breast cancer model. The EMT6-hHER-2 breast cancer cells expressing human HER2 is unresponsive to both trastuzumab and T-DM1 treatment. Our in vivo data strongly support the anti-tumoral efficacy of the T-PNU ADC as a single dose administration resulting in a complete ‘cure’ in more than 80% of tumor bearing animals. For therapeutic efficacy, HER2-targeting of the PNU payload is strictly required as neither trastuzumab, free PNU or mitoxantrone alone were able to prevent tumor growth. These results fully support the concept that by modifying the linker technology and improving the type and potency of the payload, it will be possible to generate novel ADC based therapies, which are capable of overcoming the limitations of current standard of care treatments.

Although anthracyclines are known as ICD inducers, the identification of ICD properties for T-PNU was essential to fully characterize the immune-modulatory properties of our novel compound. In this regard Rios-Doria J et al. recently reported that two different pyrrolobenzodiazepine and tubulysin-based ADCs affect tumor growth through ICD [32] leading to potent anti-tumor effects due to the stimulation of the immune system.

The clinical successes of immunotherapeutic strategies, such as checkpoint blockers, has shifted the general focus from simple drug-tumor cell interactions towards the more complex drug-tumor-immune cell interactions. However, investigation of the interplay between cancer and immune cell poses a considerable challenge owing to the evolving and heterogeneous nature of these two multicellular systems. There is a need to further dissect these complex interactions to identify predictive biomarkers, develop novel drugs, and provide mechanistic insights to support clinical decision making. We therefore performed a comprehensive characterization of the “Cancer Immunome” on the gene, gene set, gene set network, cell type and TCRβ clonotype level by next-generation mRNA sequencing. This allowed us to identify the major immunological contributors to T-PNU’s anti-tumor efficacy. T-PNU treatment-responding samples consistently clustered apart from non- or low-responsive untreated control, trastuzumab- and T-DM1-treated samples when assessed for overall gene expression, immune signatures, immune pathways and TCR clonality. In particular, an immune phenotype gene panel displaying upregulated genes of the adaptive arm of the immune system indicated a critical role of T cells in the T-PNU response. Gene set and pathway analysis revealed a particular T cell and inflammatory response in the high-responding T-PNU samples. These results suggest that targeted T-PNU therapy elicits profound changes in the tumor environment that allows homing and activation of T cells resulting in a strong cytotoxic anti-tumor response resulting in the induction of a long-lasting and potent anti-tumor immunity. The significance of CD8^+^ T cells in the establishment and maintenance of anti-tumor immunity was conclusively demonstrated by CD8^+^ T cell depletion in tumor re-challenge experiments which completely abrogated the anti-tumor immunity induced by T-PNU treatment. The involvement of CD8+ T cells and the formation of a tumor cell type specific immunological memory in tumor re-challenge experiments differs from immune-checkpoint inhibitor blockade, which generally activates a T cell response and which can lead to systemic auto-immune related side effects that may require discontinuation of treatment. The tumor-type specific anti-tumor immunity holds the promise that such unwanted side effects cause by a systemic and general activation of T cell responses by Immune checkpoint inhibitors may not occur upon translation into clinical applications.

Antibodies targeting the PD1-PDL1 axis have substantial clinical activity in different tumors and mediate durable tumour remissions [33]. Yet, only a minority of patients benefit from PD-(L)1 blockade, likely those whose tumors are pre-infiltrated by T cells. Here, our data strongly support a T cell dependence and the efficacy of the combination with PD1 blocking antibodies is achieved through non-redundant but complementary mechanisms: T-PNU augments T cell infiltration into the tumor by inducing tumor-specific, adaptive anti-tumor immunity, whereas PD1 blockade reinvigorates exhausted T cells. Similar therapeutic benefit has been observed with other chemotherapies [19, 20, 34]. Importantly, using suboptimal yet therapeutically active doses of T-PNU, our data further suggest that checkpoint inhibitors are capable to boost the anti-tumor activity of ADCs while these sub-curative doses may lead to a better tolerability of ADCs. Most notably, it can be expected that the synergistic effects of T-PNU ADCs with immune-checkpoint inhibitors may lead to a reduction of the dosing regimen of both therapeutics modalities below the effective doses of each of the drugs when used in mono-therapy. This can be expected to lead to a reduction of dose-limiting toxicities of the individual treatments.

In conclusion, this report describes the mechanism of anti-tumor immunity and long-lasting tumor protection of the novel T-PNU ADC composed of the backbone antibody trastuzumab site-specifically conjugated to a derivate of the highly potent anthracycline PNU-159682 by a non-cleavable peptide linker. Strikingly, in a breast cancer model unresponsive to trastuzumab and T-DM1, T-PNU is capable of overcoming the resistance by inducing a strong anti-tumor potency in vitro and efficacy in vivo. Furthermore, the immunostimulatory properties of T-PNU profoundly reshape the transcriptional and immunogenomic profile within the tumor micro-environment leading to the development of a long-lasting anti-tumor immunity and immune protection from subsequent tumor challenge. Finally, enhanced anti-cancer activity through combination with α-PD1 treatment hints to a promising strategy to render tumors sensitive to checkpoint blockade therapy by treatment with ADCs carrying an anthracycline payload.

## Additional file

Additional file 1: **Figure S1** Generation of a murine EMT6-hHER2 breast cancer cell line. **A**, Human-HER2 expression in EMT6-hHER2 or EMT6-WT tumor cell line determined by FACS. **B**, H&E (top panel) and 〈-hHER2 (bottom panel) immunohistochemical staining of orthotopic EMT6-WT and EMT6-hHER2 tumors. **C**, Cell proliferation assay showing EMT6-WT and EMT6-hHER2 growth inhibition after T-PNU or T-DM1 treatment in vitro. **Figure S2** Trastuzumab, free PNU or mitoxantrone treatment is ineffective in promoting EMT6-hHER2 tumor growth inhibition. **A**, Therapeutic response of EMT6-hHER2 tumors exposed to T-PNU (1 mg/kg, 2x), free PNU (6 and 8 μg/kg, 2x), trastuzumab (20 mg/kg, 4x, once per week) and mitoxantrone (4 mg/kg 1x). **B**, Survival curves of **A**. **Figure S3** T-PNU samples cluster apart from untreated, trastuzumab and T-DM1 treated samples based on gene expression. **A**, Sample similarity in a 2D projection by multi-dimensional scaling. Only the top 1000 differentially expressed genes (DEGs) are taken into account. **B**, Heatmap of the gene expression vs sample matrix. Displayed are custom selected genes plus the top 1000 DEGs (rows) of all samples (columns). **Figure S4** Heatmap PID CD8 TCR downstream pathway. **Figure S5** Gene signatures of innate immune and cytokine pathways. **A**, Network cluster of gene sets with overlapping genes with shared function of inflammatory pathway. The network includes 36 gene sets and 7551 genes (see **Table S2**). **B-D**, Heatmaps of BIOCARTA inflammatory, dendritic cell (DC) and cytokine pathway, respectively. Asterisks denote low-responding T-PNU samples. **Figure S6** Characterization of intratumoral T cells upon treatment. *p ≤ 0.05, **p ≤ 0.01. **A**, MvA plot depicting expression and effect size of selected key immune genes. **B**, Validation by FACS of selected CD8 T cell markers of functional activation and proliferation identified in **A** in between the comparisons. **Table S1** Gene sets of network TCR pathway. **Table S2** Gene sets of network activated TLR pathway. (PDF 10859 kb)
