# Supplementary material for: A novel anti-HER2 anthracycline-based antibody-drug conjugate induces adaptive anti-tumor immunity and potentiates PD-1 blockade in breast cancer
Source: J Immunother Cancer. 2019 Jan 21;7:16. doi: 10.1186/s40425-018-0464-1 (PMC6341578; doi:10.1186/s40425-018-0464-1)
Supplement: Supplementary file 1 — Figure S1 Generation of a murine EMT6-hHER2 breast cancer cell line. A, Human-HER2 expression in EMT6-hHER2 or EMT6-WT tumor cell line determined by FACS. B, H&E (top panel) and 〈-hHER2 (bottom panel) immunohistochemical staining of orthotopic EMT6-WT and EMT6-hHER2 tumors. C, Cell proliferation assay showing EMT6-WT and EMT6-hHER2 growth inhibition after T-PNU or T-DM1 treatment in vitro. Figure S2 Trastuzumab, free PNU or mitoxantrone treatment is ineffective in promoting EMT6-hHER2 tumor growth inhibition. A, Therapeutic response of EMT6-hHER2 tumors exposed to T-PNU (1 mg/kg, 2x), free PNU (6 and 8 μg/kg, 2x), trastuzumab (20 mg/kg, 4x, once per week) and mitoxantrone (4 mg/kg 1x). B, Survival curves of A. Figure S3 T-PNU samples cluster apart from untreated, trastuzumab and T-DM1 treated samples based on gene expression. A, Sample similarity in a 2D projection by multi-dimensional scaling. Only the top 1000 differentially expressed genes (DEGs) are taken into account. B, Heatmap of the gene expression vs sample matrix. Displayed are custom selected genes plus the top 1000 DEGs (rows) of all samples (columns). Figure S4 Heatmap PID CD8 TCR downstream pathway. Figure S5 Gene signatures of innate immune and cytokine pathways. A, Network cluster of gene sets with overlapping genes with shared function of inflammatory pathway. The network includes 36 gene sets and 7551 genes (see Table S2). B-D, Heatmaps of BIOCARTA inflammatory, dendritic cell (DC) and cytokine pathway, respectively. Asterisks denote low-responding T-PNU samples. Figure S6 Characterization of intratumoral T cells upon treatment. *p ≤ 0.05, **p ≤ 0.01. A, MvA plot depicting expression and effect size of selected key immune genes. B, Validation by FACS of selected CD8 T cell markers of functional activation and proliferation identified in A in between the comparisons. Table S1 Gene sets of network TCR pathway. Table S2 Gene sets of network activated TLR pathway. (PDF 10859 kb) [file 40425_2018_464_MOESM1_ESM.pdf]

Supplementary Material

Supplementary Figure 1.

Generation of a murine EMT6-hHER2 breast cancer cell line. **A**, Human-HER2 expression in EMT6-hHER2 or EMT6-WT tumor cell line determined by FACS. **B**, H&E (top panel) and α-hHER2 (bottom panel) immunohistochemical staining of orthotopic EMT6-WT and EMT6-hHER2 tumors. Images were acquired at 400X magnification. **C**, Cell proliferation assay showing EMT6-WT and EMT6-hHER2 growth inhibition after T-PNU or T-DM1 treatment *in vitro*.

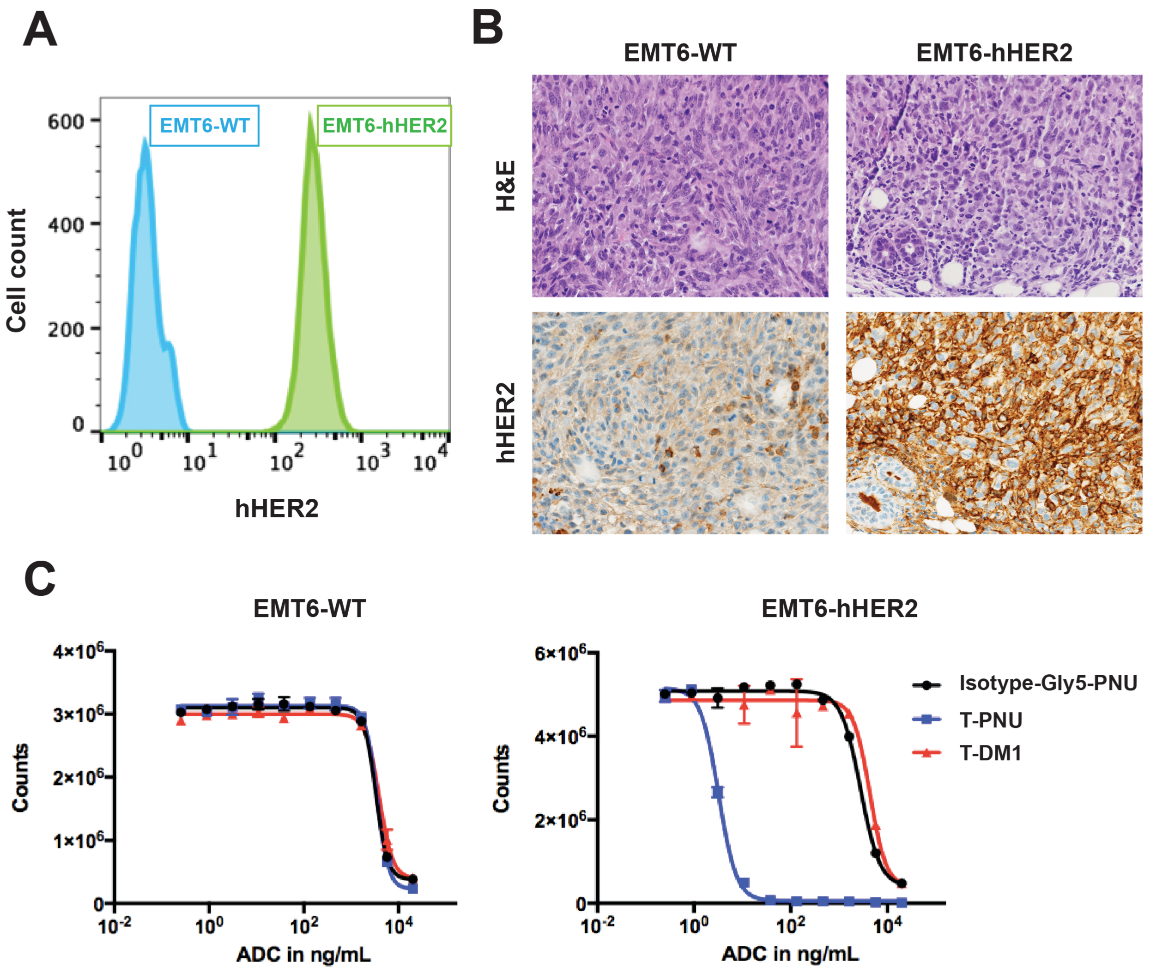

Supplementary Figure 2.

Trastuzumab, free PNU or mitoxantrone treatment is ineffective in promoting EMT6-hHER2 tumor growth inhibition. **A**, Therapeutic response of EMT6-hHER2 tumors exposed to T-PNU (1 mg/kg, 2x), free PNU (6 and 8 *μ*g/kg, 2x), trastuzumab (20 mg/kg, 4x, once per week) and mitoxantrone (4 mg/kg 1x). One representative experiment is shown (n=number of cured mice out of treated animals as indicated). **B**, Survival curves of **A**.

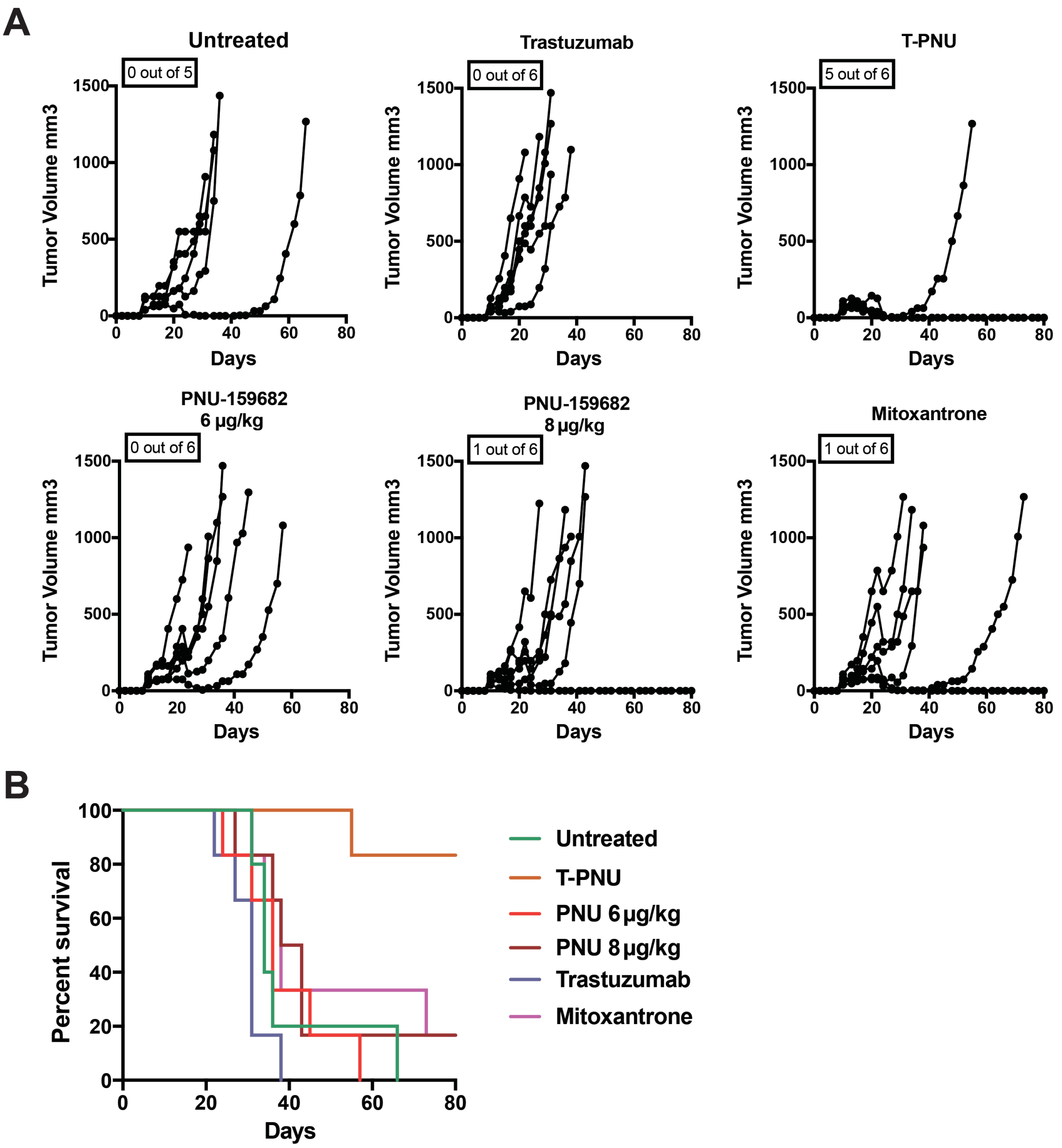

Supplementary Figure 3.

T-PNU samples cluster apart from untreated, trastuzumab and T-DM1 treated samples based on gene expression. **A**, Sample similarity in a 2D projection by multi-dimensional scaling. Only the top 1,000 differentially expressed genes (DEGs) are taken into account. **B**, Heatmap of the gene expression vs sample matrix. Displayed are custom selected genes plus the top 1,000 DEGs (rows) of all samples (columns).

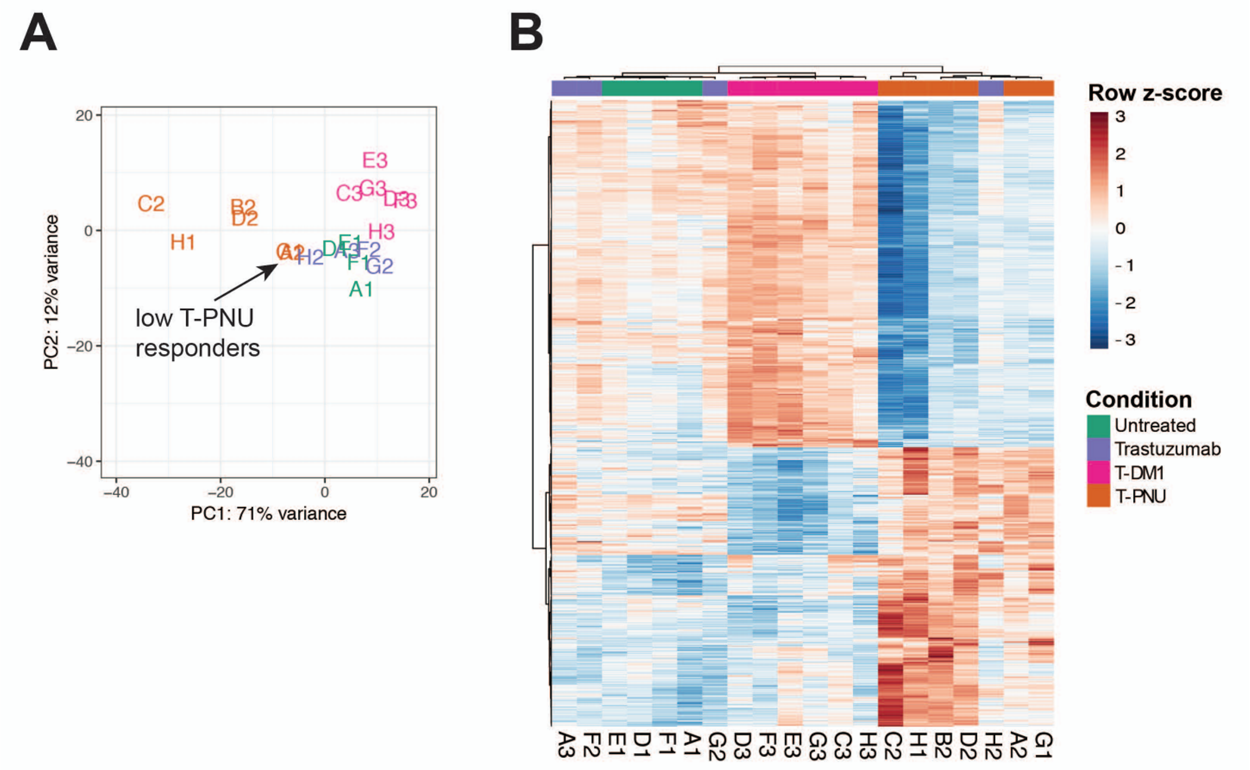

Supplementary Figure 4.

Heatmap PID CD8 TCR downstream pathway.

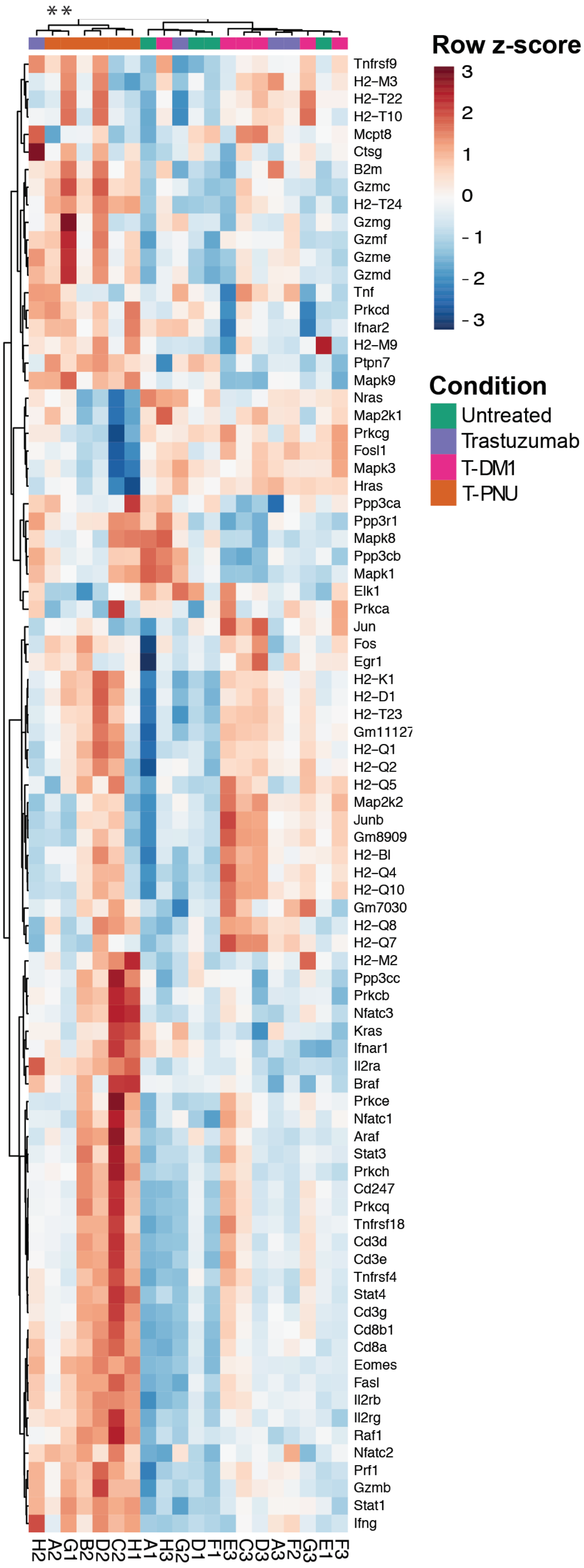

Supplementary Figure 5.

Gene signatures of innate immune and cytokine pathways. **A**, Network cluster of gene sets with overlapping genes with shared function of inflammatory pathway. The network includes 36 gene sets and 7,551 genes (see Supplementary Table S2). **B-D**, Heatmaps of BIOCARTA inflammatory, dendritic cell (DC) and cytokine pathway, respectively. Asterisks denote low-responding T-PNU samples.

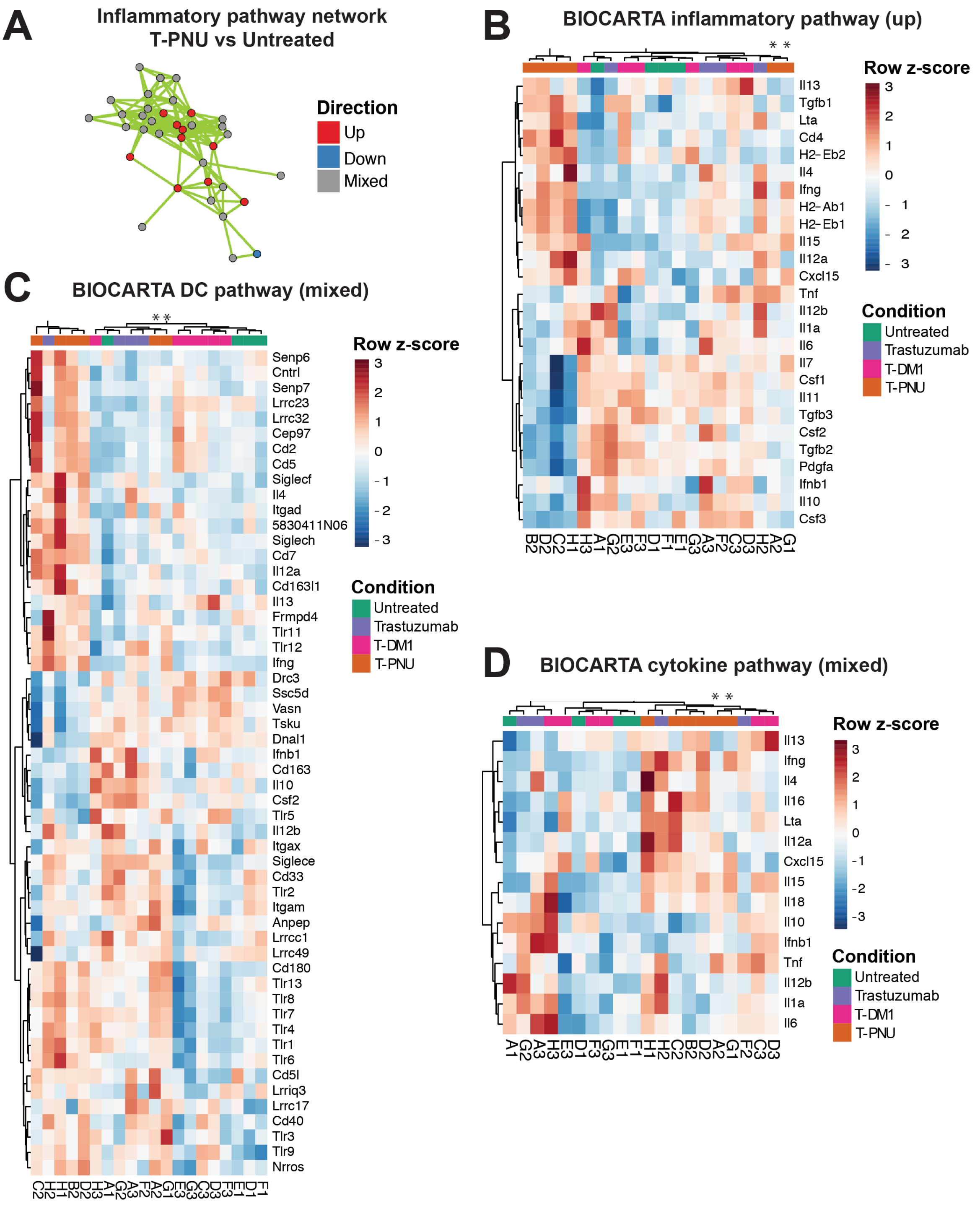

Supplementary Figure 6.

Characterization of intratumoral T cells upon treatment. *p≤0.05, **p≤0.01. **A**, MvA plot depicting expression and effect size of selected key immune genes. For each comparison, the fold-change is set in relation to the average expression of each gene. The labels indicate genes of interest. **B**, Validation by FACS of selected CD8 T cell markers of functional activation and proliferation identified in **A** in between the comparisons.

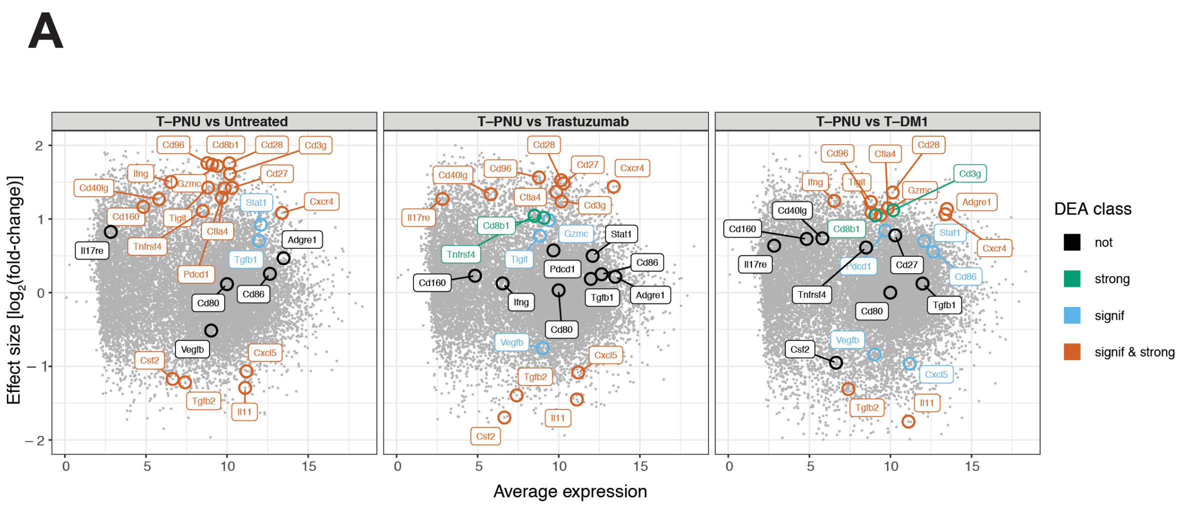

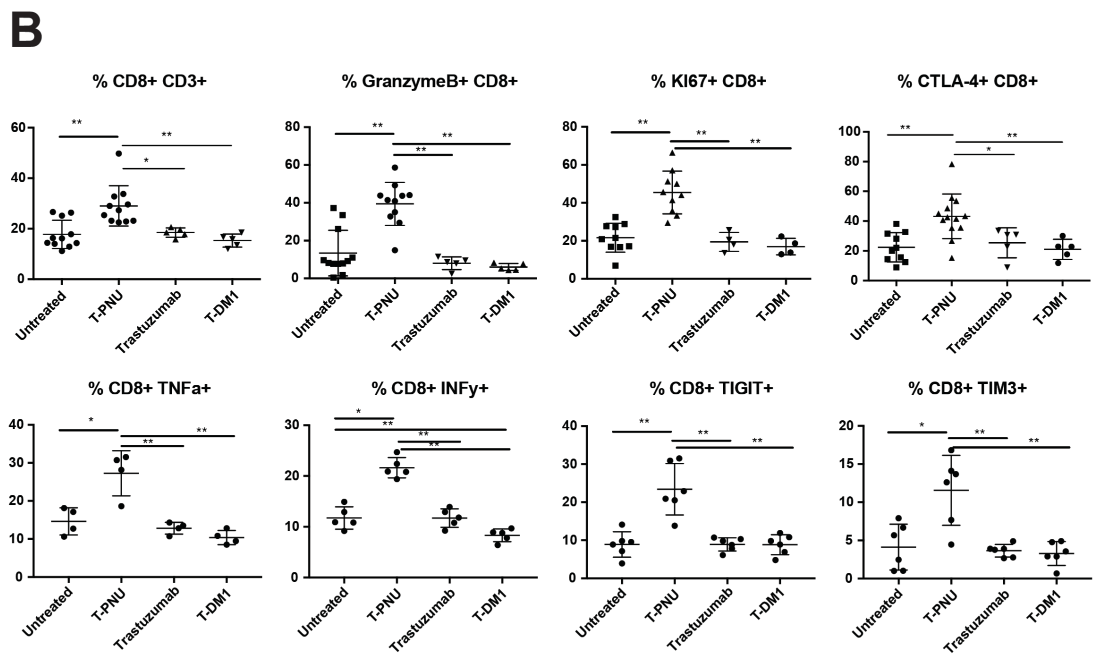

Supplementary Table 1.
Gene sets of network TCR pathway.

Name	NGenes	Direction	FDR.Mixed
BIOCARTA TCRA PATHWAY	36	Up	1.4E-05
REACTOME COSTIMULATION BY THE CD28 FAMILY	126	Mixed	2.5E-05
REACTOME CD28 CO STIMULATION	69	Mixed	3.5E-05
REACTOME CD28 DEPENDENT VAV1 PATHWAY	39	Mixed	1.2E-04
BIOCARTA CTLA4 PATHWAY	39	Mixed	5.2E-04
REACTOME CD28 DEPENDENT PI3K AKT SIGNALING	54	Mixed	5.3E-05
REACTOME REGULATION OF SIGNALING BY CBL	39	Mixed	2.3E-04
REACTOME TCR SIGNALING	105	Up	8.9E-06
REACTOME DOWNSTREAM TCR SIGNALING	69	Up	8.4E-06
REACTOME PHOSPHORYLATION OF CD3 AND TCR ZETA CHAINS	30	Mixed	7.0E-07
REACTOME PECAM1 INTERACTIONS	39	Mixed	4.4E-04
REACTOME TRANSLOCATION OF ZAP 70 TO IMMUNOLOGICAL SYNAPSE	27	Up	8.0E-07
REACTOME GENERATION OF SECOND MESSENGER MOLECULES	48	Up	1.5E-06
REACTOME PD1 SIGNALING	36	Up	8.0E-07
BIOCARTA CDC42RAC PATHWAY	39	Down	1.4E-05
PID EPHA2 FWD PATHWAY	60	Mixed	4.4E-06
BIOCARTA IL7 PATHWAY	45	Mixed	3.1E-04
PID ECADHERIN KERATINOCYTE PATHWAY	57	Mixed	1.6E-04
PID EPHRINB REV PATHWAY	75	Up	3.6E-05
REACTOME CTLA4 INHIBITORY SIGNALING	57	Mixed	1.4E-04
PID NFKAPPAB ATYPICAL PATHWAY	51	Mixed	4.3E-05
PID NETRIN PATHWAY	81	Mixed	7.0E-06
PID EPHA FWDPATHWAY	75	Up	6.3E-05
REACTOME REGULATION OF KIT SIGNALING	36	Mixed	1.5E-03
BIOCARTA EPHA4 PATHWAY	33	Mixed	2.1E-05
REACTOME DCC MEDIATED ATTRACTIVE SIGNALING	33	Mixed	4.8E-05
PID NEPHRIN NEPH1 PATHWAY	57	Mixed	7.7E-05
PID SYNDECAN 2 PATHWAY	114	Mixed	2.5E-05
BIOCARTA TCAPOPTOSIS PATHWAY	18	Up	9.0E-07
BIOCARTA SALMONELLA PATHWAY	27	Down	4.4E-05
BIOCARTA ACTINY PATHWAY	39	Down	4.1E-06
PID NECTIN PATHWAY	84	Mixed	6.4E-05
PID P38 ALPHA BETA PATHWAY	66	Mixed	3.4E-05
PID CDC42 PATHWAY	171	Mixed	1.0E-04
PID ARF6 PATHWAY	87	Mixed	1.1E-04
PID ECADHERIN NASCENT AJ PATHWAY	117	Mixed	6.5E-05
REACTOME NETRIN1 SIGNALING	57	Mixed	5.8E-05
BIOCARTA CBL PATHWAY	30	Mixed	1.1E-03
BIOCARTA RAC1 PATHWAY	54	Down	3.0E-06
BIOCARTA RHO PATHWAY	81	Mixed	6.6E-05
PID ECADHERIN STABILIZATION PATHWAY	105	Mixed	1.5E-04
PID NCADHERIN PATHWAY	102	Mixed	6.0E-05
PID RAC1 PATHWAY	129	Down	1.3E-05
REACTOME DSCAM INTERACTIONS	24	Down	6.1E-05

Supplementary Table 2.

Gene sets of network activated TLR pathway.

Name	NGenes	Direction	FDR.Mixed
BIOCARTA NFKB PATHWAY	57	Mixed	8.9E-05
BIOCARTA TOLL PATHWAY	84	Mixed	4.8E-05
REACTOME TAK1 ACTIVATES NFKB BY PHOSPHORYLATION AND ACTIVATION OF IKKS COMPLEX	39	Mixed	3.2E-05
REACTOME TRIF MEDIATED TLR3 SIGNALING	147	Up	3.9E-05
REACTOME NFKB AND MAP KINASES ACTIVATION MEDIATED BY TLR4 SIGNALING REPERTOIRE	135	Up	2.9E-05
REACTOME MYD88 MAL CASCADE INITIATED ON PLASMA MEMBRANE	156	Up	2.8E-05
REACTOME ACTIVATED TLR4 SIGNALLING	171	Mixed	3.8E-05
BIOCARTA IL1R PATHWAY	81	Mixed	3.5E-05
BIOCARTA NTHI PATHWAY	63	Mixed	3.0E-05
BIOCARTA STRESS PATHWAY	63	Down	1.5E-04
REACTOME TRAF6 MEDIATED INDUCTION OF NFKB AND MAP KINASES UPON TLR7 8 OR 9 ACTIVATION	156	Mixed	2.7E-05
KEGG TOLL LIKE RECEPTOR SIGNALING PATHWAY	177	Mixed	1.7E-05
BIOCARTA RELA PATHWAY	42	Mixed	7.3E-05
REACTOME RIP MEDIATED NFKB ACTIVATION VIA DAI	36	Mixed	1.0E-04
BIOCARTA CD40 PATHWAY	48	Mixed	8.7E-05
BIOCARTA TNFR2 PATHWAY	57	Mixed	7.3E-05
PID TOLL ENDOGENOUS PATHWAY	27	Down	6.3E-04
REACTOME MAP KINASE ACTIVATION IN TLR CASCADE	99	Up	2.4E-05
REACTOME TRAF6 MEDIATED NFKB ACTIVATION	45	Mixed	1.9E-04
BIOCARTA 41BB PATHWAY	39	Mixed	4.9E-05
REACTOME MAPK TARGETS NUCLEAR EVENTS MEDIATED BY MAP KINASES	66	Mixed	3.8E-05
REACTOME TRAF6 MEDIATED IRF7 ACTIVATION	33	Mixed	4.2E-04
REACTOME TRAF6 MEDIATED IRF7 ACTIVATION IN TLR7 8 OR 9 SIGNALING	21	Mixed	1.0E-04
REACTOME TRAF6 MEDIATED INDUCTION OF TAK1 COMPLEX	21	Up	5.6E-03
BIOCARTA RNA PATHWAY	36	Mixed	4.9E-05
BIOCARTA TALL1 PATHWAY	42	Mixed	1.9E-04
REACTOME NUCLEAR EVENTS KINASE AND TRANSCRIPTION FACTOR ACTIVATION	54	Mixed	2.8E-05
REACTOME ERK MAPK TARGETS	45	Mixed	1.0E-05
REACTOME IL1 SIGNALING	81	Mixed	7.4E-06
KEGG RIG I LIKE RECEPTOR SIGNALING PATHWAY	93	Mixed	1.4E-04
BIOCARTA DC PATHWAY	57	Mixed	6.1E-05
PID NFKAPPAB CANONICAL PATHWAY	45	Up	3.6E-04
REACTOME ACTIVATION OF IRF3 IRF7 MEDIATED BY TBK1 IKK EPSILON	21	Mixed	6.5E-04
BIOCARTA CYTOKINE PATHWAY	30	Mixed	2.7E-04
REACTOME NOD1 2 SIGNALING PATHWAY	48	Mixed	4.8E-05
REACTOME NUCLEOTIDE BINDING DOMAIN LEUCINE RICH REPEAT CONTAINING RECEPTOR NLR SIGNALING PATHWAYS	69	Mixed	3.1E-05
KEGG CYTOSOLIC DNA SENSING PATHWAY	81	Up	1.1E-04
BIOCARTA INFLAM PATHWAY	48	Up	1.3E-05
PID CD40 PATHWAY	81	Mixed	3.3E-05
REACTOME TRAFFICKING AND PROCESSING OF ENDOSOMAL TLR	39	Mixed	5.7E-05
REACTOME ERKS ARE INACTIVATED	27	Up	1.1E-06
REACTOME ACTIVATED TAK1 MEDIATES P38 MAPK ACTIVATION	24	Mixed	2.2E-04
PID IL1 PATHWAY	84	Up	2.3E-05
PID TNF PATHWAY	111	Mixed	1.1E-05
REACTOME JNK C JUN KINASES PHOSPHORYLATION AND ACTIVATION MEDIATED BY ACTIVATED HUMAN TAK1	24	Up	3.6E-04
KEGG NOD LIKE RECEPTOR SIGNALING PATHWAY	102	Up	1.3E-04
KEGG PATHOGENIC ESCHERICHIA COLI INFECTION	111	Mixed	5.6E-05
KEGG LEISHMANIA INFECTION	138	Mixed	2.1E-05
REACTOME REGULATION OF IFNA SIGNALING	27	Mixed	1.9E-05
REACTOME RIG I MDA5 MEDIATED INDUCTION OF IFN ALPHA BETA PATHWAYS	99	Mixed	2.2E-04
BIOCARTA TID PATHWAY	63	Up	8.7E-05
BIOCARTA GSK3 PATHWAY	75	Mixed	1.5E-05
REACTOME PLATELET SENSITIZATION BY LDL	42	Mixed	1.3E-04
REACTOME TRAF3 DEPENDENT IRF ACTIVATION PATHWAY	27	Mixed	2.0E-04
REACTOME NFKB ACTIVATION THROUGH FADD RIP1 PATHWAY MEDIATED BY CASPASE 8 AND10	21	Mixed	7.9E-04
BIOCARTA ATM PATHWAY	57	Mixed	1.6E-04
BIOCARTA RANKL PATHWAY	48	Mixed	5.8E-05
BIOCARTA EPONFKB PATHWAY	45	Up	2.2E-05
BIOCARTA ARENRF2 PATHWAY	18	Mixed	7.0E-04
BIOCARTA STEM PATHWAY	18	Up	2.5E-05
REACTOME CTNNB1 PHOSPHORYLATION CASCADE	36	Mixed	4.7E-05
REACTOME ADVANCED GLYCOSYLATION ENDPRODUCT RECEPTOR SIGNALING	15	Mixed	8.3E-04
REACTOME THE NLRP3 INFLAMMASOME	21	Mixed	5.0E-05
REACTOME INFLAMMASOMES	24	Mixed	3.5E-05
BIOCARTA ERYTH PATHWAY	27	Up	3.0E-04
PID TCR JNK PATHWAY	27	Mixed	6.1E-05
REACTOME IRAK1 RECRUITS IKK COMPLEX	18	Mixed	2.9E-03
